# South Africa’s protracted struggle for equal distribution and equitable access – still not there

**DOI:** 10.1186/1478-4491-12-26

**Published:** 2014-05-08

**Authors:** Hendrik CJ van Rensburg

**Affiliations:** 1 Centre for Health Systems Research & Development, University of the Free State, PO Box 339, Bloemfontein, South Africa

**Keywords:** South Africa, HRH policies, reforms, shortages, access, distribution, retention, inequalities, disparities

## Abstract

The purpose of this contribution is to analyse and explain the South African HRH case, its historical evolution, and post-apartheid reform initiatives aimed at addressing deficiencies and shortfalls. HRH in South Africa not only mirrors the nature and diversity of challenges globally, but also the strategies pursued by countries to address these challenges. Although South Africa has strongly developed health professions, large numbers of professional and mid-level workers, and also well-established training institutions, it is experiencing serious workforce shortages and access constraints. This results from the unequal distribution of health workers between the well-resourced private sector over the poorly-resourced public sector, as well as from distributional disparities between urban and rural areas. During colonial and apartheid times, disparities were aggravated by policies of racial segregation and exclusion, remnants of which are today still visible in health-professional backlogs, unequal provincial HRH distribution, and differential access to health services for specific race and class groups.

Since 1994, South Africa’s transition to democracy deeply transformed the health system, health professions and HRH establishments. The introduction of free-health policies, the district health system and the prioritisation of PHC ensured more equal distribution of the workforce, as well as greater access to services for deprived groups. However, the HIV/AIDS epidemic brought about huge demands for care and massive patient loads in the public-sector. The emigration of health professionals to developed countries and to the private sector also undermines the strength and effectiveness of the public health sector. For the poor, access to care thus remains constrained and in perpetual shortfall.

The post-1994 government has introduced several HRH-specific strategies to recruit, distribute, motivate and retain health professionals to strengthen the public sector and to expand access and coverage. Of great significance among these is the NHI Plan that aims to bridge the structural divide and to redistribute material and human resources more equally. Its success largely hinges on HRH and the balanced deployment of the national workforce.

Low- and middle-income countries have much to learn from South African HRH experiences. In turn, South Africa has much to learn from other countries, as this case study shows.

## Introduction

In recent years, as the so-called global human resources for health (HRH) crisis has escalated and the search for feasible solutions intensified, a large body of literature has accumulated on the topic of this paper. Based on this knowledge, useful conceptual, explanatory and strategic frameworks have been developed to assist in understanding HRH generally, and with a view to devising strategies to deal with both global and country-specific HRH challenges and crises [1-4]. Much of this knowledge is applicable to South Africa and at the country’s disposal for informing strategies to deal with its own HRH challenges. These include efforts to address absolute and secondary staff shortages, unequal distribution of available resources, retention of scarce resources and, ultimately, to secure the best possible coverage and access for its population to effective and equitable care.

Though aspects of the so-called global HRH crisis are apparent in all countries, the extent and seriousness of their manifestation vary from country to country. In broad strokes the World Health Organisation (WHO) [1] illustrates the variable manifestation of the crisis thus: only 10% of the global burden of disease occurs in the Americas (including Canada and the USA), yet almost 37% of the world’s health workers live in this region and more than 50% of the world’s financial resources for health are spent here. In contrast, the African region suffers more than 24% of the global burden of disease, but has access to only 3% of health workers and to less than 1% of the financial resources. The HRH situations in 36 sub-Saharan African countries have been depicted as ‘countries with critical shortage (note, South Africa is not considered to be one of these). The fact is that insufficient numbers of health workers and their unequal distribution - along public-private, rural -urban, primary-tertiary and poor-rich lines - constitute important barriers to the improvement of health outcomes and the ability of countries to meet the health-related millennium development goals (MDGs) [1,2].

One should realise however, that the worldwide HRH crisis is not only about numbers and shortfalls of health workers; rather it plays out in several dimensions. First, the crisis indeed relates to health worker shortages, but it also has to do with skills deficits and gaps in skills mixes in the workforce. Second, it refers to shortfalls in managerial and supervisory capacity, and with shortcomings in competencies to optimally deploy, utilise, support and motivate the available health workers by effective human resource management (HRM) and quality management (QM) tools to create working environments that would enable them to achieve their personal, professional and organisational goals [3]. Third, the HRH crisis involves the preparation and fitness of the health workforce to meet prevailing care demands and needs, that is, the suitability of basic and pre-service training of new entrants to core and allied health professions, and also the need for re-training, in-service and continuous training of existing personnel to enable them to deal with rapidly changing health care demands. Fourth, to these we could add that effective leadership and stewardship are necessary to consolidate these HRH essentials into a coherent whole, and then to steer it towards desirable goals. In some way or another, all countries - rich and poor, developed and developing - are confronted with these HRH challenges.

The purpose of this article is to present the case of HRH challenges in current South Africa, though thoroughly informed by the protracted history of the country’s health system and the evolution of its HRH subsystem. The article also aims to communicate details of post-1994 reforms in the country’s broader health system and its HRH subsystem to the international community, and more specifically, to highlight and convey useful lessons from the South African experience to other countries grappling with similar HRH challenges and constraints with a view to inform and direct policies and strategies to deal with country-specific challenges. Note however, that HRH dispensation in South Africa is still in the making and will be so for many years to come.

The methodology and research methods used in this research mainly comprised Internet searches for literature and research studies related to the HRH theme - including historical, comparative and factual analyses of global and country-specific HRH situations. Being a South African case study on HRH, the author concentrated in his research on South African-specific policy and legal documents, research reports, critical evaluations, and statistical reports describing and analysing the dimensions, trends and constraints of HRH from different angles, and as published by various official, professional and research bodies.

## Review

### HRH challenges - a note on African countries

Amid pervasive poverty and meagre material resources, African countries generally experience serious HRH constraints and shortfalls that result in precarious provisioning of and access to Western or allopathic health care for their populations. The HRH situations of sub-Saharan Africa are characterised by poorly developed health professions and a dearth of tertiary training institutions to produce health professionals. Moreover, the available health professionals are concentrated in urban centres, which leave vast rural areas and remote populations either underserved or unserved. Low levels of staff motivation due to a lack of equipment, frequent shortfalls of supplies, high vacancy rates and mounting workloads appear to be - after chronic staff shortages - the second most important workforce problem in African countries, especially in rural areas and facilities [3,5]. Few African countries have comprehensive HRH policies and plans, and even where they do, funding does not follow for implementation. Issues of retention and remuneration thus remain unresolved [2]. For Dovlo [4], HRH in African countries are also disturbingly plagued by wastage - both direct and indirect - which in many ways erodes the numbers, capacity and productivity of the health workforce. In addition, there is a constant, debilitating drain of the already meagre resources to more developed countries that offer better living standards, working conditions and career advancement. This serves to exacerbate the weaknesses and further undermines the already failing African health care systems. Ultimately, it seriously threatens the achievement of health equity in the region [6]. Retention policies, incentive schemes and restrictive measures of whatever kind, inter-country agreements, financial restitution, ethical guidelines and human rights considerations - all of these fail to stem such constant haemorrhaging of the meagre supply of HRH (especially doctors and nurses) in African countries [2,6]. As a result, a close relationship has developed, on the one hand between absolute and secondary HRH shortages and poor HRM in African countries, and, on the other, the prevailing, mostly preventable diseases and deaths in their populations. The general shortage of health workers, combined with poor HRM could thus be taken to figure among the main constraints to achieving the health-related MDGs in African countries [3,7].

Because of this scarcity of conventional health professionals and the resultant constrained access to Western-type health care, many African countries are necessitated to resort to home-grown models of care or to consult their own varieties of traditional healers so as to enable them to cope with either the lack of or the backlogs in access to care, especially access to health care of a professional and specialised nature. After Alma Ata in 1978, primary health care (PHC) was strongly advocated and established in many developing countries. It introduced new approaches to health care and shifted the emphasis in care to new types of health workers. Several such innovative HRH strategies and care models thus originated and/or gained firm footholds in African countries in efforts to satisfy the ever-escalating demand for health care and the severe shortages of qualified HRH. Among these strategies were decentralising care to peripheral facilities, substituting professional workers with lower-level professionals and non-professionals, task-shifting and task-sharing among health workers [8-10].

These developments have resulted in an upsurge of various types of single- and multi-skilled community health workers (CHWs)- as community-based extensions of health services – though differently named and with different functions in different countries: lay carers, counsellors and supporters, health extension workers, health surveillance assistants, village health workers, home visitors, rural midwives and expert patients, whose primary role it is to perform preventive and basic curative services as part of comprehensive PHC [2,3,5,10,11]. Some of these HRH innovations (in both personnel and care models) have gained prominence and have expanded rapidly during the HIV/AIDS epidemic, which resulted in immense pressure on the meagre professional HRH establishments of weak health systems - a situation once referred to as the twin or double burden of the HR and HIV crises in sub-Saharan Africa [5]. Research in a number of sub-Saharan countries has convincingly demonstrated the unavoidable necessity of task-shifting and also the benefits of task-shifted care models for increased programme efficiency, productivity and cost-effectiveness, expanded access and coverage, the quality of care, health outcomes, and team dynamics in HIV care, thereby allaying some of the reservations about quality and safety [9-12]. However, HIV/AIDS also generated huge HRH wastage in all its manifestations: deaths and illness among health workers, fear of infection, heavy workloads, absenteeism, stress, burnout, demotivation, low morale, loss of productivity, public-sector attrition and the exodus to other countries [4].

Of course, the HRH developments affect the conventional health professions in that both the scopes of the professions and the scopes of practices have had to be redefined. Ultimately, these trends imply de-professionalisation of medicine, nursing and other core health professions. These HRH innovations however, have also shown that conventional, Western-type health professionals and care models are often a luxury in deprived areas in Africa due to enormous shortages and incumbent costs for populations.

### South Africa’s HRH challenges, constraints and strategies

South Africa’s health care system, its HRH establishment and the supply of health professionals do not mirror the dire shortages and constraints found in other African countries. South Africa has strongly developed health professions, and in most categories a relative abundance of health professionals and of mid-level and auxiliary workers linked to the core professions [13-16]. With 4 doctors, nurses and midwives per 1000 population, South Africa’s HRH provisioning falls well above the WHO ‘critical’ benchmark of 2.5 health workers per 1000, and also far above those of most other African countries [1,17]. Still, the country’s HRH are plagued by problems and constraints similar to those being experienced by other developing countries. These include marked HRH shortages, inappropriate skills mixes, unequal distribution of available personnel, a disturbing exodus of health professionals to developed countries, and severe public-to-private drainage, rural-to-urban migration, and poor-to-wealthy settings. As a result, health care in South Africa was (and still is) differentially and unequally apportioned to the population along public-private, rural -urban, poor-wealthy, state dependent-medically insured lines, while some of these divides still carry racial overtones.

The combined result of this differential apportioning is that by far the majority of South Africans have limited and severely constrained access to health care. They often receive care of lesser quality due to heavy patient-loads at overburdened, understaffed and ill-equipped public health facilities. In contrast, the wealthy, self-paying and medically insured population (the minority by far) has abundant access to large numbers of private practitioners and facilities that concentrate in urban areas, and which lavishly deliver easily accessible care of superior quality. Similar contrasts and constraints - sparse distribution of health workers, constrained access, less sophisticated facilities, and care of lesser quality - typify health services for rural and poor populations.

### HRH distribution and access to care during South Africa’s colonial and apartheid eras

Past and present socio-political (especially racial) and socio-economic (both privatisation and socialisation) policies played and are still playing pivotal roles in the structuring of the South African health system, the organisation and deployment of HRH and in access to health care. The impacts of racial policies - early colonialism, apartheid and separate development - led to a race-based division of health services into separate institutions, later aggravated by health services being organised into separate homelands, each with an own health department and own professional bodies. These policies impacted heavily on the historical maldistribution of HRH and on differential access to health services [18]. Race-based exclusion from the health professions and from the mainstream training institutions for health professionals as such prevailed for many decades. Only in the last years before the dismantling of apartheid were professional membership and training institutions made generally accessible to all races. At a similarly late stage, the policy of race-segregated care institutions was terminated and care was provided to all races in health facilities. The important outcomes of this protracted racial exclusion and segregation on the HRH side of the health system were that the majority of health professions developed along stark racial lines marked by white privileging and dominance, further distorted by male dominance. Huge backlogs in the numbers of health professionals from the deprived race groups accumulated, especially in the ranks of the black South Africans. On the patient side of the health system, deprived subpopulations (mainly non-whites) had to be satisfied with a smaller supply and an inferior quality of care provided in racially segregated, poorly equipped and understaffed public facilities.

As for socio-economic policies, the South African health system from its inception embarked on a two-pronged course. On the one hand, private practitioners and private care institutions were from the outset part of a split or two-class health system. At times, white governments of the day boosted the privatisation of health care. This was the case especially during the 1970s and 1980s, when health care was made the responsibility of the individual and was apportioned according to one’s ability to pay [18]. Even after 1994, health care became increasingly privatised, as was illustrated in the mushrooming of sophisticated private-for-profit hospitals and the already strong yet steadily growing contingent of health practitioners then flocking to the private health sector. For the more privileged, especially white clientele, health care was financed by numerous third parties, that is, health insurance companies co-financed by privately paying members and their employers. At the other end, patients who were not medically insured had to depend on the public health services with the state footing the bill, or had to pay themselves or make do without care. As already said, public sector services were always limited by constrained material and human resources, with crippling effects on both access and quality of care rendered to the majority of the population.

From this deep private-public divide in the national health system the distorted distribution of HRH arose, manifesting in both overprovision and under-provision of staff and coverage: abundant, even superfluous access and universal coverage as against limited, constrained and even no access and coverage. The end result is a two-class system of health care: more and of better quality for the wealthy and the privileged; less and of poorer quality for the impoverished and the deprived. These exclusionary and discriminatory policies of the past rendered South Africa an ‘extreme example of inequity’ in every sphere of life, including health care [19]; it left post-apartheid South Africa with a health system ‘profoundly and explicitly inequitable’ [20], and characterised by unjust distribution of resources and unequal capabilities and rights [21].

### HRH in post-apartheid South Africa – redistributing resources and broadening access

The transition of South Africa to full democracy in 1994 introduced fundamental reforms both in broader society and its health system. The Constitution of 1996 promoted an egalitarian health care system and a break with the legacy of gross inequality and discrimination [22,23]. To realise these goals, the African National Congress (ANC)-ledgovernment pursued a series of pro-poor and pro-equity policies and legislation [24,25]. Regarding HRH specifically, the post-apartheid government successfully introduced a number of reform strategies with a view to rectifying core HRH problems of the time. These included the multiple forms of maldistribution of health personnel, skewed race and gender profiles in staffing, inequities in training health professionals, reorienting health workers towards PHC, and strengthening the public health sector via various staff-focused interventions [16].

During the first years of post-apartheid South Africa, major strides have been made towards establishing a just health dispensation, more equal distribution of financial and human resources for health, and more equitable access to care. The free-health policies and various priority programmes went a long way towards broadening access to care for the most deprived and vulnerable groups. The introduction of the district-based PHC system, the reprioritisation of the health budget so as increasingly to favour PHC, and the building and upgrading of numerous PHC facilities, were all responsible for distributing HRH more equally and also rendered health care more accessible to the poor and to people living in rural areas [26]. Affirmative policies made significant progress towards race and gender representivity in public staff establishments and in the health professions student corps [16]. Collectively, these initial reforms represented a first wave of post-1994 health reforms [27].

Nonetheless, during these first years of a democratic South Africa, the HRH scene remained disconcertingly unchanged in important respects. HRH were still strikingly unequally deployed between the public and the private sectors (Table 1), and were geographically still highly unequally distributed along provincial and rural -urban lines (Table 2). Approximately 70% of all general medical practitioners - 86% of dentists, 76% of pharmacists and 93% of psychologists - were quite recently still deployed in the non-public/private sector [13]. Similarly, remnants of the historical race discrepancies and backlogs are today still reflected, for example, in the numbers of medical practitioners (including specialists): 16 936 (whites), 8354 (African blacks), 5314 (Indian) and 927 (coloured) [14]. Access to health care is, for example, still differentially allotted to the wealthy (privately insured, about 16% of the total population) and the poor (non-insured/state-dependent, 70% plus), while the main race groups are still disproportionally covered by medical insurance (8,9% African black, 20,3% coloured, 41,1% Indian and 69,7% whites) [14]. These disparities in care provisioning obviously create concomitant disparities in access to care and ultimately also in the health profiles of the different subpopulations. In 2002, huge health disparities were still related to race, gender and age as was exemplified in infant mortality rates that varied between 7 per 1000 for whites and 67 per 1000 for blacks, while life expectancy at birth for white women was 50% longer than for black women. There were also relentless increases in the under-five mortality (from 59 per 1000 in 1998, to 104 per 1000 in 2007), maternal mortality (369 per 100 000 in 2001 to 627 per 100 000 in 2007) and young adult deaths (especially young women - four times higher in 2005 than in 1997) [28-31].

**Table 1:** Public sector and non-public sector distribution of selected health professionals (2010)

**Sector**		**Public sector**		Non-public sector*		**Public/non-public ratio**
**Professional category**	**Total registered**	**Number**	**%**	**Number**	**%**	
Medical practitioners	36 912	11 309	30.6	25 603	69.4	1:2.2
Dentists	5320	770	14.5	4550	85.5	1:5.9
Pharmacists	12 218	2966	24.3	9252	75.7	1:3.1
Physiotherapists	5777	1009	17.5	4768	82.5	1:4.7
Occupational therapists	3508	838	23.9	2670	76.1	1:3.1
Psychologists	7037	498	7.1	6539	92.9	1:6.6
Registered nurses	115 244	51 966	45.1	63 278	54.9	1:1.2

*The category, non-public sector, should not simply be equated to private-sector health professionals. While it does indeed include private health professionals deployed in the private (for-profit) health sector, it also includes many health professionals who are retired, not actively practising or working overseas, and also health professionals deployed in the non-governmental organisation (NGO), faith-based organisation (FBO) and other private-not-for-profit sectors.

**Table 2:** Provincial disparity ranges (only extremes) of selected health professionals in the public sector (2010)

**Professional category**	**Provincial disparity range (best and worst ratios)**
Medical practitioners	Western Cape 1:737 versus 1:5805 Limpopo
Registered nurses	Free State 1:512 versus 1:1191 Gauteng
Dentists	Western Cape 1:4854 versus 1: 42 496 Limpopo
Pharmacists	Western Cape 1:3532 versus 1:15 812 Limpopo
Physiotherapists	Western Cape 1:3 855 versus 1:39 704 Limpopo
Occupational therapists	Western Cape 1:6301 versus 1:65 319 North West
Dental therapists	Mpumalanga 1:54 810 versus 1:1 741 302 Western Cape
Psychologists	Gauteng 1:3568 versus 1:53 328 Limpopo

The post-1994 reforms have thus largely failed to effect sufficient change in HRH distribution and in access to health care. Specifically women, children and the aged from the poorest echelons and from rural areas, continue to live less protected from health hazards, are more exposed to diseases of poverty, have to rely on health services of compromised quality, and have less access to services as a result of poverty-related barriers. In 2004, Padarath *et al*. [32] observed that there was no strategic human resources (HR) plan in health to bring the divergent HR components into a coherent framework, that HR policy remains *ad hoc*, and that HR planning for the future is largely wanting. Where such plans did and do exist, for example, the consecutive HRH plans of 2006 [33] and 2011 [34], their systematic implementation for various reasons either lags behind or fails [16]. Quite dishearteningly the Department of Health recently observed: ‘The leadership, processes and data have not been in place for effective health workforce planning. The lack of planning results in an unmanaged health workforce, where attrition, shortages, poor access, and dissatisfaction become part of the culture of health professionals in the South African health system [34].

### Public-private disparities in HRH distribution and access to care

The essence and extent of public-private (or public-non-public) disparities in HRH distribution and the resultant differential access to different types of care boil down to the following: Although South Africa spends a rather high 8.6% of its gross domestic product (GDP) on health, this figure hides the approximate 44% (4.1%) to 56% (4.5%) proportional division between the public and the private sectors, a skewed allocation aggravated by the fact that only a small minority of the population is covered by private health insurance [35-38]. Some years ago, McIntyre and Van den Heever [39] illustrated the public-private disparities in HRH distribution as follows: ‘… each pharmacist in the public sector serves 12 to 30 times, and each generalist doctor in the public sector 7 to 17 times, more people than those in the private sector (depending on whether one focuses only on the medical scheme population or assumes that up to 35.8% of the population use private pharmacists and general practitioners). There is a six fold difference in the number of people served per nurse, and a 23 times difference in the number of people served per specialist doctor, working in the public and private sectors in South Africa. These access inequalities are determined by the differential affordability of care to different subpopulations and various direct and indirect cost factors that either facilitate or constrain/limit access for different socio-economic (read also racial) sections of the population. The majority of black Africans (75.5% or 25.2 million) and more than half (56.1% or 3.1 million) of coloured people rely on the public health sector - about 74% of the total population. In contrast, 83.4% (3,6 million) of whites and 65.5% (970 000) of Indians have access to the well-resourced private health sector [37]. Ataguba and McIntyre [40,41] and McIntyre *et al*. [35] have recently concluded that in general the distribution of health benefits (in both the public and the private sectors) are not only pro-rich, but also not in line with the need for health care; richer groups receive a far greater share of service benefits even though they carry a relatively lower share of the illness burden. Table 1 details the public and non-public distribution of selected health professionals in South Africa [13,16].

In all the selected health professional categories (nurses excluded) pronounced distributional disparities present between the public and the non-public/private sectors - provision overwhelmingly favouring non-public/private sectors, though varyingly so. The smallest public/non-public disparity ratio is for medical practitioners (range 1:2.2), whereas the ratio for psychologists (range 1:6.6) is the most extreme. Expressed differently, almost 70% of medical practitioners work in the private/non-public sector, whereas almost 93% of all psychologists are deployed in this sector. In contrast, nurses in South Africa are primarily employed in the public sector, especially in provincial health services (mainly hospitals and PHC facilities). However, during the past two decades the number of nurses employed in the private/non-public sector has steadily increased - the recent figure being around 42%, a doubling of the 21% figure of two decades ago [34,42]. This trend is attributable to the expansion of the private hospital industry during this time, the increased employment of nurses in the private sector, and the accelerated production of nurses by private nurse-education institutions for deployment and retention in private facilities [43]. It is remarkable that nurses in the public sector serve approximately six times more people (1:616) than do those in the private sector (1:102) [44].

Other sources indicate, for example, that for the 10-year period between 1997 and 2006, there was a decline of 25% (n = 854) in the number of specialists in the public sector, whereas medical practitioners in this sector increased by a mere 8.43% (from 9184 to 9958) [16,45]. Such disparities (and public-sector deficits) are, inter alia, perpetuated in the low absorption/retention rates of new graduates in the public sector: over a 10-year period, only 30% of all new graduates have been retained in the public sector, that is, only 4403 (37,6%) of the 11 700 medical doctors; only 248 (11.8%) of the 2104 dentists; and fewer than 20% of physiotherapists and occupational therapists [34]. Over the longer term, it appears that the public-private (non-public) mixes of several professional categories have changed marginally, in general towards a slight strengthening of public-sector components (percentages deployed in the public sector for 1999 followed by those for 2010): general practitioners (27.4% to 30.6%; dentists (7.4% to 14.5%); pharmacists (23.7% to 24.3%); physiotherapists (13.6% to 17.5%); occupational therapists (19.5% to 23.9%); psychologists (5.8% to 7.1%) [13,16,46]. Presently, as in the past, nurses (all categories) represent the majority by far (around 80%) of South Africa’s total health workforce; they also provide the bulk of public-sector health services. South Africa’s health system is therefore primarily nurse-based and nurse-driven [34].

Public-non-public/private disparities deepen drastically when superimposed on the existing provincial disparities. The extent of these combined provincial/public-private disparities is aptly illustrated in the following figures: in 2007, the more affluent Western Cape had 60 private hospitals, 55 public hospitals, and 1246 doctors for a population of 4.8 million, whereas the less affluent Limpopo had 6 private hospitals, 44 public hospitals and 882 doctors for a population of 5.7 million [20].

### Geographical (rural -urban and provincial) disparities in HRH distribution and access to care

Worldwide, HRH have for many years been distributed unequally between urban and rural areas. In South Africa, this kind of disparity can be translated to more urban and to more rural provinces in that some provinces are predominantly urban, others overwhelmingly rural. The favourable supply of health professionals in the public sectors of certain provinces is further attributable to the proportions of medically insured - as opposed to state-dependent patient populations - residing in the different provinces, and also to the large concentration of private practitioners, private hospitals and tertiary/academic complexes found in some provinces. The rural HRH deficits are clearly illustrated in the following: although 43.6% of South Africa’s population reside in rural areas, they are served by only 12% of the country’s doctors and 19% of its nurses [37]. The magnitude of the unequal HRH distributions (more favourable as against less favourable) in the public health sector becomes clear when one compares the disparity ranges (best and worst ratios) in the distribution of public health professionals in the nine provinces – in Table 2 only expressed in terms of the provincial disparity extremes [16].

Geographical disparities of this nature are constantly reinforced and aggravated by the movement of health professionals, especially from rural areas to urban centres. Lehmann’s [47] analyses of 2008 further articulate examples of this particularly South African HRH dynamic, namely that the interprovincial disparities in the distribution of medical doctors and professional nurses in the public sector have over the longer term significantly decreased - the doctor/population ratio in Limpopo has become more favourable (from 6 to 17.4/100 000), while in Western Cape there has been a marked deterioration (from 50 to 33.8/100 000). For the country as a whole, there has been a slight deterioration from 25 to 24.4/100 000. In contrast, the supply of professional nurses in the public sector has deteriorated drastically both in provinces and also in South Africa as a whole: in Limpopo (from 212 to 115.3/100 000), Western Cape (from 269 to 114.0/100 000) and for the country (from 251 to 110.4/100 000). Furthermore, the urban/more affluent provinces – in contrast to the rural/less affluent provinces – are home to large proportions of specialists in the public sector: in 2008, 71.8% of all public-sector dental specialists were based in Gauteng, while in 2010, medical specialists in Gauteng (38.7%) and Western Cape (28.7%) together accounted for two-thirds (67.4%) of all medical specialists in the public sector. Such markedly favourable distributions of specialists in the public sectors of two provinces were most probably ascribable to the large academic hospital complexes and the huge medically insured populations in the said provinces. What is certain is that the geographical distribution of the health workforce determines what services will be available, and also their quantity and their quality. Imbalances cause problems of equity (services not being available according to needs) or efficiency (surpluses/shortages) and of effectiveness of services, not to mention the satisfaction of users [2].

### Internal and external drain of HRH - public to private and South Africa to other countries

A further HRH matter of serious concern in South Africa is the constant brain-drain, either from the public to the private sector or via emigration to other countries. In the latter case, there is a continuous and sizable exodus of health professionals to developed countries, especially to Britain, Canada, the US, Australia and New Zealand, thus eroding the national stock of professionals and aggravating existing imbalances in distribution and access to services. Some time ago, it was estimated that health professionals leave South Africa at a rate of 25% per year [34] and that 37% of South Africa’s medical practitioners and 7% of its nurses have migrated to other countries [44,48]. The reasons for this country-to-country migration hinge on the strength of various push/pull factors and stick/stay factors (material and non-material incentives/disincentives) within South Africa as a source country (and its health care sector) and in the recipient country (and its health care sector) [15-17,49].

Similarly, the two main forms of with in-country migration of health professionals, that is, rural to urban and public to private, are enduring problems in that these workforce flows constantly cause and aggravate existing imbalances and inequalities both in the distribution of HRH and also in access to care; they thus weaken both public and rural health services, while also negatively affecting the effectiveness of services [17]. This migration is generally explained in terms of the public health sector being fraught with push factors as over the strong pull factors that draw health professionals to an attractive private (both for profit and not for profit) health sector with its resource-rich environment, apparently better working conditions and career opportunities, attractive remuneration packages and reward/incentive schemes [15-17,44]. The trend is further fuelled by the as yet booming private hospital and health care industry, the expansion of the private hospital sector to countries abroad, the rise of private training institutions for nurses that supply nurses to the private sector, and the upsurge of private nurse agencies that contract nurses in the public sector for moonlighting, and, similarly, specialists for dual practice [17,50]. In contrast, public HRH establishments are often plagued by staff shortages that are aggravated by the chronicity of vacant posts, budget constraints, deteriorating infrastructure, equipment and services, down sizing of staff establishments, freezing of posts, and the resulting job insecurity, unfavourable and unsatisfactory working conditions, poor remuneration, lack of career progression prospects, dual practice (moonlighting) by public health professionals, low staff morale and job dissatisfaction among public-sector professionals, and, an absence of effective strategies to retain personnel [15,17,31,33,50]. Many of these reasons also resonate with those given for the non-recruitment/non-absorption of new graduates in the public service. In the case of medical doctors, these reasons culminate in a lack of policy to augment the numbers, a lack of planning, a lack of both finances and posts, inadequate working environment and conditions, very limited and even non-existent career prospects in the public health services, and also the absence of positive reinforcement: ‘Doctors often feel undervalued, and some policy and financing incentives support this perception. The South African health and education system have, by omission or commission, implemented “push factors” which send doctors away [15,51].

The general effect of the loss of health professionals to other countries and also to the private sector is that imbalances and inequalities are generated in the country’s supply and distribution of HRH. These imbalances and inequalities create HRH shortages, scarcity of skills and deficient skills mixes, which then give rise to disparities and inequities in access to care and in health coverage. Ultimately, these deficits render the health system ineffective, the quality and outcomes of health care are compromised, and so, in the final analysis, is people’s health [16]. The loss of health professionals to other countries also means a huge monetary loss in terms of investments made in the training of health professionals. This loss further places a heavy burden on the limited resources available for the production of yet more health professionals to compensate for these losses.

### Strategies for promoting efficiency, access, redistribution and retention

Since 1994, the government has launched various HRH production, distribution and retention strategies in an effort to better balance existing public-private and urban -rural disparities and to strengthen both its public and rural health services. These strategies aim at retaining HRH in under-resourced areas and in public health facilities, and at deploying the workforce more equally and according to need, so as to expand both access and coverage. Recently, two HRH-specific plans for South Africa were launched to address the spectrum of recurring HRH challenges, and detailed strategies, among others, to step up the production and recruitment of health professionals, to steer their distribution/redistribution, and to retain them in the public sector and in the country [33,34]. Both plans, however, tend to be rather overambitious in their HRH projections, and both seem to lack the necessary follow-up implementation [16]. About the same time, three further strategic policy documents of relevance for HRH followed: the *HRH Strategy for the Health Sector* (2012-2017) [52], the more comprehensive *National Health Insurance Plan*[53] and the even broader society-based and long-term strategic *National Development Plan*[54]. All these are continuations and extensions of the previous HRH policy and strategy documents. Indeed these documents again emphasise, from different angles, the very same broad and specific HRH challenges and constraints, and similar interventions to overcome these. They are all geared towards dealing with the serious human resources shortages, meeting the escalating demand for health care, addressing the uneven and often poor-quality public health services, ensuring equity of access and universal coverage, scaling up health worker training, increasing the supply of health workers, improving their productivity, and retaining them in the workforce, and closing the gaps between human resources in the public and private health sectors. In essence, the above policies and plans are all forward -looking frameworks that keep a huge unfinished business in the health sector on the HR reform agenda. They also reaffirm the vision and direction already explored since 1994. Besides their strategic importance, however, it remains a crucial question whether the notorious policy-implementation gap (or plan-action disconnect) could be effectively bridged. As indicated elsewhere in this article, and in so many commentaries on South African health policies and plans, the government’s success record in this respect is generally less promising.

Past and present recruitment, distribution, redistribution and retention strategies have pursued - though with varying effectiveness and variable sustainability - the following [16,32-34,44,55-57]: (1) to introduce affirmative student recruitment (race and gender) for admission to tertiary training and the preferential recruitment of quotas of candidates from rural areas with a view to later deploying them in these areas; (2) to recruit and import foreign health professionals (originally especially Cuban doctors, and later also, through agreements between governments, from several other countries) to serve especially in rural areas and in designated public health facilities; (3) to introduce various financial incentive schemes framed in rural allowances and scarce-skills allowances (at a time respectively applying to 33 000 and 62 000 full-time health professionals) to address the dual private-public and rural -urban inequity in the distribution of health professionals; (4) to introduce an occupation-specific dispensation, a later incentive-driven strategy to improve service conditions and the remuneration of health professionals so as to attract and retain them in the public sector; (5) to increase the intake of students at tertiary training institutions in order to increase the numbers of professionals and mid-level health workers; (6) to train clinical associates (a mid-level medical category) to serve as substitutes for medical doctors in poorly resourced areas and facilities; (7) to train contingents of South African students as doctors in Cuba eventually to be deployed in the public health sector; (8) to introduce a national CHW policy framework that makes provision for the availability of generalist CHWs attached to PHC facilities; (9) to permit public-sector health professionals also to enter via dual practices and moonlighting into private-sector services; (10) to contract private general practitioners into the public service to deploy them in under-resourced public facilities to enhance public PHC services; (11) to encourage public-private partnerships so as to improve the managerial efficiency, the financial sustainability and the quality of public health care delivery, and to recruit and retain health workers in the public sector; (12) to issue Certificates of Need to rationalise the utilisation and promote the more equitable allocation and distribution of health delivery and of HRH in the country; and (13) to introduce compulsory community service (CS) for all health professionals once they have completed their training.

The distribution and retention strategy that has had the strongest impact on countering HRH shortages and on the maldistribution of HRH in the South African public health sector has probably been the introduction of compulsory CS: this was first introduced in 1998 for doctors and since then systematically expanded to cover all graduating health professionals, with the last of these being the nurses in 2008. The primary aim of CS is to distribute health personnel more equitably with a view both to making health services more accessible and alleviating the unequal rural -urban and public -private distribution of health professionals [51,58]. Its long-term, positive effect is that it annually strengthens the public health sector by injecting large numbers of health professionals into this sector - from 1998 to 2010 more than 30 000 - including 13 155 doctors (since 1998), 1812 dentists (since 2000), 4068 pharmacists (since 2001) and 4605 nurses (since 2008) [16]. In 2012 alone, 7162 health professionals were placed across all provinces [57]. The negative effects of CS as a measure for distributing and retaining HRH have also been recorded, such as that few CS professionals have stayed on in public service and that the system serves as a strong push factor for young professionals to emigrate [15,51,56,58].

It is nevertheless perturbing that several of the above strategies are neither regularly nor systematically evaluated. Evidence is thus lacking regarding the effectiveness and gains of some of these strategies for motivating, recruiting, distributing and retaining HRH. Obviously, some have thus, in the course of time, been stalled, phased out and shelved or shown to have a limited effect on distributing, retaining and motivating staff.

### HRH in the time of HIV/AIDS – emergent models and cadres of care to broaden coverage and access

The post-1994 reforms of South African society and its health system coincided with the emergence and escalation of HIV and AIDS. For two decades, the epidemic has been exerting huge and progressive pressure on both the material resources and the health workforce of the country. The government’s early approaches to the epidemic- especially its initial denial, and since 2004 also its centralised, vertical and overcautious approach to antiretroviral treatment (ART)- resulted in slow and constricted roll-out and scale-up and, in turn, in limited and constrained access [59]. Recent estimates indicate that 5 786 603 South Africans are currently living with HIV, more than 300 000 are annually infected, while, in 2011, an estimated 1 793 000 people received ART (1 525 000 in the public ART programme and respectively 190 000 and 78 000 in the private and NGO sectors). Treatment coverage increased from 7% in 2003 to 84% in 2010 [14,59,60]. These figures indicate the magnitude of the scourge and of the immense backlogs in coverage that have over many years built up in the numbers of people in need of ART. In recent years, larger government allocations, a drop in drug prices, decentralisation of the programme, relief from restrictive guidelines and cumbersome administrative burdens of the ART programme, nurse-initiation of treatment and task-shifting, and the lifting of treatment eligibility criteria to 350 cells/μl, all opened up and broadened access to HIV care [59]. The positive effects of this expansion of access are evident in the decreasing AIDS mortality and the increasing life expectancy [38,59].

Against this backdrop, the epidemic obviously lays heavy, increasing and multiple burdens on HRH [31,44,61-64]. First, health care workers (especially those serving in public health facilities) have to shoulder the impacts of a rapidly increasing disease burden in the general population as growing patient numbers give rise to increasing demands for health services and extraordinary workloads for and pressure on the often lean staff establishments of public health facilities. Second, health care workers themselves have to cope with increased morbidity and mortality in their own ranks as significant numbers fall ill or succumb to the disease, thereby reducing the supply of health providers through deaths, absence from work and reduced performance. In such cases, the remaining staff members have to take on increased workloads. Third, HIV/AIDS and its concomitants, that is, fear of infection in the workplace, the effects of the disease on those living with AIDS and the negative effects on staff morale, all increase stress and burnout among health workers, often resulting in their quitting the public health sector and emigrating.

The growing demands thus made on HRH by the epidemic and the concomitant need for appropriate and affordable care have forced South African policy makers and managers - in line with many sub-Saharan countries - not only to reconsider the human resources and skills needed for coping with the epidemic, but also to lighten the heavy workloads of the core professions and, at the same time, to expand access and improve the effectiveness of health services. In particular, the epidemic induced them to contemplate options wider than and beyond the conventional professional cadres, and moreover to consider non-conventional models of care and novel cadres of health carers.

In South Africa as in other African and poor-resourced countries much has indeed materialised during the era of HIV/AIDS and ART scale-up that has stimulated the emergence of task-shifted care models and the utilisation of various forms of health worker substitution. Task-shifting, task-sharing and skills delegation involve transferring tasks from qualified professionals to mid-level and even lay cadres of health workers who have shorter training and fewer qualifications. Doing this serves to reinforce, increase and optimally utilise the health workforce in an attempt to cope with expensive HR shortages and skills deficits, the ultimate aim being to improve access to HIV care and other health services [8,9,12,64,65]. It is the shortage of health professionals at the core of the public-sector ART programme - doctors, professional nurses and pharmacists - that renders substitution by mid-level and lower level workers not only inevitable but also necessary. When properly implemented and managed, health-worker substitution and task shifting can indeed contribute in major ways towards improving and expanding coverage and access to HIV services, and towards ensuring sustainable, cost-effective, accessible and equitable health care [10,66-69].

Though there are approximately 40 000 CHWs in South Africa, a key challenge facing the utilisation of these CHWs is the ‘lack of organisation and standardisation within this health worker category’ and the fact that it is a ‘largely volunteer-based and underpaid health worker category’ [44]. Despite such drawbacks, many reports testify to successes in shifting PHC responsibilities (especially the responsibilities of ART treatment, care and support) not only from doctors to nurses, but also to CHWs [70-73]. However, health-worker substitution and task shifting in HIV care and ART delivery should not be seen as a panacea for all HRH challenges. Success in this regard hinges both on being part of an overall strategy that includes measures to increase, retain and sustain health staff, and on being deployed within an enabling regulatory framework and as part of integrated health teams - thus not as a temporary add-on at the periphery of the formal health system [10,12,66,68]. The WHO [65,74] therefore calls for checks and balances, because health worker substitution, task-shifting and task-shifted models represent a radical departure from conventional delivery models that depend on specialist workers and should thus be implemented alongside other efforts to increase the numbers of skilled (professional) health workers.

The introduction and multiplication of these substitute workers have significantly and most probably permanently changed the face of HRH in South Africa as they increasingly take on functions and roles that are normally the reserve of internationally recognised health professionals. Such developments are necessarily accompanied by the redefining of both the scopes of conventional health professionals and their scopes of practices, and they inevitably contain elements of de-professionalisation of the conventional health professions.

### A second wave of post-1994 health reforms - reprioritisation and revitalisation

The lack of progress towards a more equitable health dispensation after 1994 could be ascribed to a series of barriers. Foremost among these was the unmoveable legacy of the apartheid health system, one characterised by a strong emphasis on curative and institutional care, the favouring of private health care in government policy, and the rigidly entrenched race- and class-based divisions present within both the provider and the clientele components of the system. However, several commentators [30,31,36,38,75-84] have in recent years attributed this lack of progress to deficiencies and failures in the post-1994 reform process itself. These are summarised by Van Rensburg and Engelbrecht [25] as follows: first, the introduction of the district-based PHC system - as the main lever with which to effect an equitable health dispensation - did not live up to high expectations; second, PHC was implemented rather selectively, with little regard for the original comprehensive approach, and health was narrowly pursued as health care by government and by the health departments, while there was only limited community involvement; third, gaps or disconnects between policy/plans and policy implementation assumed chronic proportions in the public health sphere; fourth, more and more evidence was tabled that the fundamental flaws in the national health system also lay in inadequate stewardship, poor leadership and a lack of management capacity at all levels of the system; fifth, it became clear that the health system’s performance was not commensurate with the huge investments that were being made. It now seems unlikely, even impossible that most of the health-related MDGs and targets will have been attained by 2015.

Progress towards the idealised egalitarian health dispensation thus fell far short. As a result, a second wave of health reforms followed in around 2007, with the ANC National Congress (Polokwane) serving as a turning point. The government has since realised and acknowledged that the national health system was not performing as had been envisaged and hoped for at the beginning of the post-1994 dispensation. Subsequently, challenges and their causes have been defined anew and various task groups have been charged with the planning and implementation of further reformative steps with a view to revitalising and overhauling the failing national health system. Central to this agenda is the reprioritisation of the health needs of the national population to focus on the quadruple burden of disease with stronger emphasis on prevention and health promotion. Furthermore, it has once again become apparent that the transformation of the health system hinges closely on how it is structured, staffed and financed. As a consequence, several reform strategies are currently being prepared and introduced to translate the refined and redefined policies and priorities into practice [28-31,34,35,77,80,83,85-88]. These reform foci and strategies are intended to strengthen health systems, overhaul the financing of the national health system, revitalise PHC and re-engineer the district health system.

### The National Health Insurance (NHI) Plan and HRH - new prospects of balanced distribution and equitable access

Several sources capture the essence of the health reforms since 2007, and many of these reforms are embodied in the NHI Plan currently being phased in [16,37,53,57,84,88]. Historically, the underlying tenets of social health insurance (SHI)/NHI - equitable access and universal coverage in health care - came onto the South African scene in as early as the 1920s and have since regularly been resurrected and driven by various political and civic groups. In the 1940s, national health coverage was on the verge of becoming official health policy, and from 1955, the Freedom Charter made universal coverage part of the agendas of national liberation movements. However, the interests of a strong free-market conglomerate (white government, the health professions, and big business) always successfully countered such ideals [25]. With the transition to full democracy in 1994, the ANC put and subsequently kept the SHI (NHI) high on its health-reform agenda. Yet, it did not make the desired headway. A convergence of events in around 2007 was followed by firm steps to make the NHI a reality. In 2011, an NHI Committee produced the Green Paper on NHI [53]. Whereas previous attempts to introduce universal coverage were rather limited and selective in scope, the current NHI Plan aims to be comprehensive, inclusive and equitable in that it intends to cover all South Africans free of charge at the point of care [37]. It is a bold, reformative initiative calculated to eliminate disparities and inequities in health care, to effect universal coverage by distributing health resources equally, to ensure equitable access to health care, and to strengthen the under-resourced and strained public sector with a view to improving the performance of the entire health system [57]. If implemented according to the principles of the NHI, and if the necessary material and human resources could be mobilised, NHI indeed promises universal coverage and equitable access to care. It is sure not to be a smooth run; its HRH dimension, especially the staffing of underserved districts and facilities in rural areas, will indubitably pose the most substantial challenge to its realisation.

An important part of the larger NHI Plan concerns the re-engineering of PHC, which boils down to a reaffirmation of the district-PHC system as introduced after 1994. However, this time it will be accompanied by a thorough revitalisation of its core principles in order to strengthen its capacity and to broaden its reach. To a large extent, the re-engineering of PHC is simultaneously also a pervasive HRH strategy: among others, it intends fundamentally to reorganise and strengthen the prevailing HRH dispensation by multiplying and redeploying the existing health care corps, and to employ and produce new staff cadres suited to pursuing universal coverage and equitable access for all. The intention thus is to distribute and redeploy HRH within health districts, while focusing more specifically on the priority health needs of district populations, and more strongly emphasising disease prevention and health promotion, doing so in accordance with the shortfalls revealed in respect of the targets set by the health MDGs [36,83,84,89].

To this end, different models of care and different district-based health teams will be composed, strengthened and deployed. Each type of team will have far-reaching HRH consequences in that each will require variable numbers of health workers and varying skills mixes. In brief, it is envisaged that the re-engineering of PHC is planned to be provided in three streams: (1) a district-based model of specialist clinicians - obstetrician, gynaecologist, paediatrician, family physician, advanced midwife, senior primary care nurse - to provide clinical direction and specialised support to PHC clinics and outreach teams in each district; (2) a municipal ward-based PHC model constituting both clinic-based and community-outreach teams, each being composed of a part-time medical practitioner, a PHC nurse practitioner, professional or staff nurses, counsellors, nursing auxiliaries, post-basic pharmacist assistants, administrative support staff, and CHWs/community care givers (CCGs); (3) a school health programme comprising school health teams run by nurses to provide regular health education and promotion, and also preventive and screening services at schools. As in the past, the re-engineered PHC services will be nurse driven, that is, nurses will remain at the core of all three of the PHC team types, but they will now be adequately trained in PHC, and able to support and supervise mid-level and auxiliary nurses and the cadres of CHWs/CCGs. To establish these teams, staff establishments need to be redesigned; numerous new posts will have to be created and their contents defined; current scopes of practice (roles and tasks) will have to be redefined and reassigned; and, large-scale task shifting/task sharing will have to take place. In addition, large numbers of health professionals and new cadres of health workers will eventually have to be recruited, trained/retrained or deployed/redeployed to the districts with a view to staffing the PHC clinics and the community-outreach teams [16,35,53,57,84,90].

In 2012, the stepwise phasing in of the NHI commenced with the introduction of selected components of PHC re-engineering in eleven pilot districts and with representation of the nine provinces. Notable progress has been reported [54] and it is planned to implement the NHI fully within 14 years [53].

## Conclusion

Over centuries, the evolving South African health system has entertained elements both of free-market and state-provided health care. This resulted in a deep structural split between a public health sector (state-provided health care to a majority clientele of state-dependent people) and a private health sector (privately provided health care to a minority clientele of wealthy people). Successive governments sporadically reinforced this divide by means of policies advancing either state-provided or free-market health care. The health workforce also became divided into those health workers serving in the public sector as state employees as over those providing health care in the private sector as private practitioners or entrepreneurs. During the colonial and apartheid dispensations an additional structural dimension was superimposed on this public-private structure: through policies and legislation of racial exclusion, segregation, and white privileging, the health workforce was developed and deployed according to race. One result was that huge race-related backlogs accumulated in the production of health professionals. Similarly, huge disparities and inequalities developed in the distribution of health workers, especially between those serving the wealthy and those serving the poor, those deployed in urban areas as over those deployed in rural areas, and between those catering for the medically insured population as over those serving people who were dependent on the state. These disparities were in the rule accompanied either by more or by lesser access to care and by better or worse quality of care.

There are no quick and easy ways to erase such structural deformities in the HRH sphere that crystallised in the course of centuries. The recent reforms of South African society and its health system, including the HRH dispensation, are bold efforts to normalise and rectify prevailing distortions and constraints in respect of access to care, inequities in distribution of resources, and differential quality of care for different population groups. At the basis of these distortions lies the structural split between the private and public health sectors, which in turn generates an array of HRH deficiencies, such as over- and under-concentrations of health personnel, under- and over-served areas and populations, over- and understaffed health facilities, draining of scarce HRH to urban areas and the private sector. Such deformities are aggravated by the absence of effective retention strategies and lack of proper staff supervision and motivation in the public health sector amid low levels of job satisfaction and staff morale among public health workers. Take note that similar conditions and constraints present in most other countries, though they are more pronounced in developing countries, including African countries.

The post-apartheid government introduced fundamental reforms with a view to promoting justice and equity in the health sphere, and indeed made commendable progress towards strengthening the public sector and broadening access to care via a series of pro-poor and pro-equity policies. However, after two decades these reforms still fall short of ridding the health system of the deeply entrenched public-private sector split and the concomitant unequal access to health care for different population groups. The available HRH still continues to be unequally distributed between the public and the private sectors, while access to health care continues to be highly inequitably distributed among rich and poor and among those living in urban areas and those in rural areas, while these often also coincide with racial affiliation. To address the severe HRH shortages in deprived areas and in vulnerable populations, a series of strategies have been implemented to produce and recruit HRH and to distribute and retain these resources. These have, however, met with variable success. In fact, South Africa has neither overcome its HRH constraints nor remedied the inequitable access to health care. Since 2007, the government has been placing its hope on an NHI Plan and accompanying strategies as a possible solution to the need to balance the distribution of the workforce and to secure universal and equitable access to health services for the entire population.

The South African case presents a good illustration of the kind, variety and intensity of HRH challenges a country could experience. At the same time, during the country’s post-apartheid search for solutions to the large variety of its own HRH constraints, South Africa generated numerous successful health-sector reform strategies which also provides excellent examples of how countries could deal with their HRH challenges, how they could steer health reforms aimed at constrained access, distribution and retention in efforts to create more equal and equitable and universally accessible health care dispensations. In all these respects, all countries, and especially developing countries, could learn important lessons from South Africa’s experiences. This means not only learning from the many successes in the country’s record of reforms in the field of HRH, but also lessons from the many failures and initiatives that went astray in this domain. Note that the purpose of this article was not only historical analysis limited to South Africa, that is, not only focusing on HRH developments in past South Africa and inside the country. The aim was also to look forward and outward, and thus to highlight future implications of HRH developments for the health system, as well as to bring South African experiences in HRH to the attention of the international forum, in particular to impart applicable and useful lessons to countries grappling with similar challenges and constraints in the HRH domain.

Generally such lessons span the entire domain of HRH, including the spheres of policy and legal development and institutionalisation; reform of the health professions and the health workforce, whether the racial and gender transformation of professional components, redirecting professional training towards priority care needs; the deployment or redeployment of existing staff establishments to serve new and redefined goals; or the training, deployment and integration of mid-level workers and CHWs into the public health system. Numerous such HRH lessons have sprung from Africa’s 20 years of health reforms, which have abundantly demonstrated major transformation successes in core areas. Lessons (both positive and negative) of particular value for HRH are exemplified in the early introduction of free-health policies to expand access to care for previously deprived and vulnerable populations; the establishment of the district-based PHC system, along with the reorientation and retraining of staff to render the system viable; the prioritisation of PHC in allocating national resources which resulted in huge gains in access to care; the reform of the health professions and the redirection of professional training; the introduction and maintenance of the largest HIV/AIDS/ART programme in the world, along with the redeployment and training staff to scale up the programme. More recently, South Africa has embarked on establishing its NHI Plan to overhaul the entire health financing system, which also implies far-reaching reorganisation of HRH and training and of staff the plan requires.

NHI is in important respects a continuation of the direction in which the health care system has developed since 1994. It also foreshadows the trends in health care both in South Africa but also internationally. NHI, if executed as planned and if the necessary material and human resources can be mobilised, indeed promises universal coverage and equitable access to care for South Africa’s entire population. It could take the country many strides further in creating equal, equitable and accessible health services for all. Surely the NHI initiative will not be a smooth run, and the outcomes of the series of NHI interventions cannot be predicted with any certainty. In particular the HRH dimension of NHI, specifically the proposed staffing of underserved districts and facilities in rural areas, will indubitably pose a most substantial challenge to its realisation. In addition, the effects of the NHI on the national health system and on the current workforce will remain uncertain for years to come. In theory, NHI will activate a new dynamic that could potentially strengthen the existing workforce, distribute it more equally, retain its members more successfully, prompt more effective public health services, and expand access to health services. However, unintended and as yet unforeseen consequences of the evolving NHI and the huge state bureaucracy it implies, could surface and just as well cripple and weaken the future workforce by setting in motion forces that may lead to opposite and undesirable outcomes, especially if the recurring lack of stewardship, leadership and management capacity apparent in the broader state machinery and specifically in the public health sector is not mended. Nevertheless, NHI for South Africa has come to set the future nature and pace for HRH in South Africa; it will generate numerous and valuable lessons - whether constructive or destructive for the national health system, and whether with positive or negative outcomes for the HRH dispensation - for the world, and especially developing countries, to take note of. These reforms put a huge unfinished business and on going HRH reforms on the HRH plate.

In light of the above and to put the message of this article in broader perspective, it is appropriate to conclude by emphasising three important reminders relevant to the theme and aptly applicable to South Africa: first, the WHO [65] acknowledges that, though the health sector can take significant actions to advance health equity, the ‘roots of health inequities lie in the social conditions outside the health system’s direct control. Second, Dussault and Franceschini [2], speaking in similar vein, remind us of the importance of this broader (read South African) context at play: ‘Ultimately, only equitable socioeconomic conditions for rural compared to urban areas, adequate investment in human resources, and stable and legitimate political institutions are the basis for achieving a balanced distribution of the health workforce. Third, Mathauer and Imhoff [3] caution us to be realistic in that:

‘Improved human resources management cannot compensate for many other factors, both at the macro and micro level, that seriously impinge upon work performance and staff retention, such as staff and supply shortages, heavy workload and difficult working conditions, migration pull factors originating from developed countries, as well as the threatening HIV/AIDS pandemic. Non-financial incentives and HRM/QM tools are not a magic bullet that solves the pressing HRH problem and compensates for the lack of investment and the structured deficits that characterise health systems in many low-income countries - there is no such magic bullet. However, these tools can make a difference and may be effective even in a resource-constrained context.

The struggle for the balanced distribution of the health workforce, for equitable access to health care, and for universal health care coverage of the entire population is clearly not over. South Africa is still not there.

## Abbreviations

ANC: African National Congress; ART: antiretroviral treatment; CCG: community care giver; CHW: community health worker; CS: community service; FBO: faith-based organisation; GDP: gross domestic product; HR: human resources; HRH: human resources for health; HRM: human resource management; MDG: millennium development goal; NGO: non-governmental organisation; NHI: National Health Insurance; PHC: primary health care; SHI: social health insurance; QM: quality management; WHO: World Health Organisation.

## Competing interests

The author declares that he has no competing interests.
